# Guidance for protocol content and reporting of factorial randomised trials: explanation and elaboration of the CONSORT 2010 and SPIRIT 2013 extensions

**DOI:** 10.1136/bmj-2024-080785

**Published:** 2025-02-04

**Authors:** Brennan C Kahan, Edmund Juszczak, Elaine Beller, Megan Birchenall, An-Wen Chan, Sophie Hall, Paul Little, John Fletcher, Robert M Golub, Beatriz Goulao, Sally Hopewell, Nazrul Islam, Merrick Zwarenstein, Diana Elbourne, Alan Montgomery

**Affiliations:** 1 MRC Clinical Trials Unit at UCL, London WC1V 6LJ, UK; 2 Nottingham Clinical Trials Unit, School of Medicine, University of Nottingham, Nottingham, UK; 3 Institute for Evidence-Based Healthcare, Bond University, Robina, QLD, Australia; 4 Women’s College Research Institute, University of Toronto, Toronto, ON, Canada; 5 Primary Care Research Centre, School of Primary Care, Population Sciences and Medical Education, Faculty of Medicine, University of Southampton, Southampton, UK; 6 *The BMJ*, BMA House, London, UK; 7 Department of Medicine, Northwestern University Feinberg School of Medicine, Chicago, IL, USA; 8 Health Services Research Unit, University of Aberdeen, Aberdeen, Scotland; 9 Oxford Clinical Trials Research Unit, University of Oxford, Oxford, UK; 10 Centre For Studies in Family Medicine, Schulich School of Medicine and Dentistry, Western University, London, ON, Canada; 11 London School of Hygiene and Tropical Medicine, London, UK

## Abstract

This report presents the explanation and elaboration paper for the CONSORT (Consolidated Standards of Reporting Trials) 2010 and SPIRIT (Standard Protocol Items: Recommendations for Interventional Trials) 2013 extensions for factorial trials. Factorial trials involve randomising participants to more than one intervention, often with the aim of evaluating multiple interventions in one study or assessing whether treatments interact. The CONSORT and SPIRIT statements have been extended to allow for the unique features of the factorial design. Reporting items along with detailed explanations and examples of good practice are provided, as well as a glossary of key terms and an overview of the methodological features of factorial trials.

The SPIRIT (Standard Protocol Items: Recommendations for Interventional Trials) 2013 and CONSORT (Consolidated Standards of Reporting Trials) 2010 statements provide checklists of reporting items to be included in trial protocols and trial reports, respectively.^1^ ^2^ ^3^ ^4^ The primary focus of these two statements are two-arm trials that use an individually randomised parallel group design.^1^ ^2^ ^3^ ^4^ Many of the reporting items in the CONSORT and SPIRIT statements apply equally to trials with other designs. However, some designs require items to be modified or added. Extensions of the CONSORT 2010 and SPIRIT 2013 statements have been published for cluster randomised trials,^5^ non-inferiority and equivalence trials,^6^ crossover trials,^7^ multiarm trials,^8^ stepped wedge trials,^9^ adaptive^10^ and early phase trials,^11^ pilot and feasibility trials,^12^ and n-of-1^13^ ^14^ and within-person trials.^15^

In a factorial trial, participants are allocated for multiple factors, each of which comprises both an intervention and its comparator. In the 2×2 trial shown in box 1, participants were allocated between eicosapentaenoic acid and placebo (factor 1), as well as between aspirin and placebo (factor 2).^16^ Factorial trials can be used for different objectives (box 2), such as evaluating multiple interventions in one trial without increasing the sample size (2-in-1 trials; box 3 and box 4) or evaluating whether interventions interact.^19^ ^20^ ^21^ ^22^ Factorial trials have additional methodological complexity compared to standard two-arm parallel group trials, including the specific trial aims, presence of interactions (box 4), choice of treatment groups used in main comparisons, and non-concurrent enrolment (box 4).^19^ ^20^ ^21^ ^22^ ^23^ ^24^ ^25^ ^26^ ^27^ ^28^ ^29^ ^30^ ^31^ ^32^ ^33^ ^34^ ^35^ ^36^ ^37^ ^38^ ^39^ ^40^ ^41^ ^42^ ^43^ ^44^ ^45^ ^46^ However, such aspects are frequently poorly reported.^30^ ^34^ ^39^ ^40^ ^41^ ^47^ Few trials provide a rationale for using the factorial design, making it challenging to determine whether appropriate study methods were used.^34^ ^41^ ^47^ In addition, those trials that do provide a rationale often use inappropriate methodology. For instance, many trials that use a factorial design to evaluate whether treatments interact do not power the trial to test this,^40^ and trials using the factorial design for efficiency frequently do not report testing for interactions—a key assumption underpinning the design.^34^ ^39^ ^41^

Box 1 **Illustration of a factorial trial**

The SEAFOOD Polyp Prevention Trial was a 2×2 factorial trial assessing eicosapentaenoic acid (EPA) and aspirin to prevent colorectal adenomas in participants with sporadic colorectal neoplasia.^16^ ^17^

The trial had two interventions (EPA, aspirin), each with a single comparator (placebo EPA, placebo aspirin). Thus, two factors and two levels existed within each factor: the EPA factor comprised of active EPA and placebo EPA, and the aspirin factor comprised of active aspirin and placebo aspirin).

**Table:** 

EPA	Aspirin	
	Active	Placebo
Active	Active EPA and active aspirin	Active EPA and placebo aspirin
Placebo	Placebo EPA and active aspirin	Placebo EPA and placebo aspirin

This trial used a full factorial design, meaning that all participants were eligible for each factor and randomised between levels of each factor (ie, all participants were randomised to one of four treatment groups: EPA alone, aspirin alone, EPA and aspirin, or double placebo).

SEAFOOD was a 2-in-1 trial, because it used a factorial design to facilitate efficient evaluation of both EPA and aspirin against their respective comparators in a single trial without tangible increases to sample size requirements. This approach was based on the assumption that the interventions would not interact—that is, the effect of EPA would be the same when used on its own or when used in combination with aspirin (and similarly for the effect of aspirin).

The trial had two main comparisons: all EPA (EPA and aspirin, EPA alone) versus all not EPA (aspirin alone, double placebo); and all aspirin (aspirin and EPA, aspirin alone) versus all not aspirin (EPA alone, double placebo). Thus, each comparison used a factorial analysis (see glossary in box 3), which relied on the assumption that the interventions do not interact.

Box 2 **Reasons to use a factorial design in a trial**

Factorial trials can be used for different reasons. Two commonly stated reasons are to efficiently evaluate two or more interventions in a single trial without materially increasing the sample size (2-in-1 trials), or to evaluate whether interventions interact. These two objectives require different methodological considerations, including:

- Choice of main comparison(s): whether the interaction is of primary interest, or the comparisons between factors.
- Sample size calculations: larger sample sizes are required to detect interactions than to compare levels within a factor.
- Choice of trial design: partial factorial designs (where some participants are not randomised for certain factors) are typically not appropriate when the primary objective is to determine whether interventions interact, because patients not randomised for specific factors cannot be included in an analysis to estimate interactions for those factors.

Owing to the different methodological requirements, these aims (efficient evaluation *v* evaluation of interactions) are generally mutually exclusive—that is, a trial cannot be designed to deal with both aims satisfactorily. A trial powered to detect an interaction would not retain the efficiency gains inherent to 2-in-1 trials, while a trial powered for efficiency would be underpowered to detect interactions.

As such, the objective in using a factorial design should be clearly defined in advance, and methodological considerations should be aligned with that objective.

Box 3 **Glossary of key terms**

**Factorial trial**

A trial in which two or more interventions are assessed in the same participants.

**Factor**

Each intervention and its comparator(s) together comprise a factor.

**Level within factors**

These levels are specific interventions and comparators within a factor.

**Treatment group**

The unique combinations of factors and levels to which participants can be randomised (eg, active eicosapentaenoic acid (EPA) and active aspirin comprise one treatment group).

**Full factorial design**

All factors and levels are combined so that the design comprises all possible combinations of factor levels, and all participants are eligible to be randomised for each factor.

**Partial factorial design**

Some participants are not randomised for certain factors.

**Fractional factorial design**

Some combinations of factors are omitted.

**2-in-1 trial**

A factorial trial designed to evaluate the effect of two or more interventions in a single trial with minimal impact to the required sample size.

**Comparison**

Which treatment groups will be compared against each other.

**Main comparison(s)**

The comparison(s) that will primarily be used to draw conclusions about effectiveness of each intervention.

**Estimand**

A description of the treatment effect to be estimated from the trial. Factorial trials must specify whether interest lies in estimating the effect of one intervention in the presence or absence of the other factor.

**Factorial analysis (or “at the margins” analysis)**

All participants allocated to active A are compared against all those allocated to control A, and similarly for the factor B comparison.

**Multiarm analysis (or “inside the table” analysis)**

Comparison of the treatment groups against each other (eg, active EPA and placebo aspirin, placebo EPA and active aspirin, etc).

**Interaction**

Occurs when the effect of one intervention depends on whether participants also receive the other intervention.

Box 4 **Methodological considerations in factorial randomised trials**

**Design**

- Research objectives:
  - Factorial trials can be used for different objectives, and methodological considerations (including type of factorial design, sample size calculations, and statistical methods) should be driven by these objectives.
- Interactions:
  - Interactions pose a key challenge for 2-in-1 trials, which require the assumption that interventions do not interact. Trials can be designed to limit the likelihood of interactions, for instance, by choosing interventions expected to work along different pathways, or are given at different time points^18^; however, interactions cannot typically be ruled out entirely in advance. Therefore, assessment of interactions forms a key component of 2-in-1 trials.
- Eligibility criteria:
  - 2-in-1 trials can use different eligibility criteria for different factors. For instance, participants who have a contraindication to factor B could still be randomised for factor A (termed a partial factorial design). However, the use of differing eligibility criteria requires additional consideration during analysis (ie, participants who were not randomised for a factor should not be included in the analysis of that factor).
  - Using different eligibility criteria across factors is usually not appropriate for trials whose primary aim is to evaluate whether interventions interact, as patients not randomised for specific factors cannot be included in an assessment of any interaction for that factor, and so do not contribute to the trial’s primary objective.
- Sample size:
  - The sample size calculation should be guided by the main trial objectives and should be large enough so that each main comparison has adequate power.
  - In 2-in-1 trials, a sample size is typically calculated for each main comparison, with the largest calculated sample size chosen if they differ across comparisons.
  - For trials whose primary objective is to evaluate an interaction, the sample size should typically be calculated on the basis of detecting an interaction with a sufficient degree of power.

**Conduct**

- Interim analyses and stopping guidelines:
  - Many trials use formal methods for interim monitoring and early stopping, because of benefit, harm, or futility. In factorial trials, either entire factors (eg, factor B) or treatment groups (eg, group A and group B) could be stopped early. This approach leads to non-concurrent enrolment of participants between factors, which must be handled appropriately during analysis.

**Analysis**

- Estimand:
  - An estimand describes the treatment effect that the trial sets out to quantify. The choice of estimand should be guided by the main trial objectives.
  - Factorial trials require additional considerations for the choice of estimand compared with other trial designs.
- Estimation:
  - The chosen analysis approach should align with the main trial objectives (estimand). In 2-in-1 trials, a factorial analysis is typically used because it takes advantage of the efficiency gains inherent to the factorial design. This analysis is predicated on the assumption that treatments do not interact.
  - Alternative analyses can be used, including a multiarm analysis. The multiarm analysis does not require the so-called no interaction assumption; however, it is underpowered compared with the factorial analysis and so is often used as a sensitivity analysis to evaluate robustness of main results to deviations from the no interaction assumption.
  - In trials where certain participants are not randomised for some factors (either because they are not eligible for that factor or because that factor has ceased recruitment due to early stopping—termed “non-concurrent enrolment”), these participants should not be included in the comparison for the factor to which they were not randomised, because their inclusion means that the analysis is no longer based on a randomised comparison.
  - For trials whose primary objective is to evaluate whether interventions interact, the primary analysis would typically involve estimating the size of the interaction, along with a confidence interval.
- Reporting and interpretation:
  - 2-in-1 trials are predicated on the assumption of no interaction, and interpretation of such trials might depend on whether interactions are identified, or thought likely based on the magnitude and precision of estimated interaction terms.
  - Evaluation of both the direction of the estimated interaction and results from sensitivity analyses (eg, from multiarm analyses) can help determine to what extent conclusions might be affected from likely interactions.
  - Even if interactions are identified, this identification does not necessarily render study results invalid. For instance, an interaction might indicate that the factorial analysis is underestimating the intervention effect, and that the true effect could be larger. This result would imply that despite uncertainty over the specific magnitude of benefit, there could be reasonable certainty that the intervention offers some benefit, despite the interaction.

To facilitate improved reporting of factorial trials, CONSORT and SPIRIT extensions for factorial trials were developed.^48^ ^49^ This explanation and elaboration paper presents each modified or new checklist item from the CONSORT and SPIRIT extensions, along with a detailed rationale and examples of good reporting. A list of terminology is provided in box 3, and an overview of methodological features is provided in box 4.

**Summary points**

Factorial randomised trials have unique complexities that require additional reporting compared to standard parallel group designs
The extensions to the CONSORT (Consolidated Standards of Reporting Trials) 2010 and SPIRIT (Standard Protocol Items: Recommendations for Interventional Trials) 2013 statements for factorial trials aim to improve the reporting of such trials
This article provides a detailed rationale for each modified item along with examples of good reporting

## Scope of this article

This explanation and elaboration paper is intended to be used in conjunction with the CONSORT 2010 and SPIRIT 2013 statements^1^ ^2^ ^3^ ^4^ as well as the extensions for factorial trials.^48^ ^49^ For clarity, the primary focus is on 2×2 factorial trials, although most of the recommendations extend to more complex factorial designs (box 5).

Box 5 **Reporting considerations for more complex factorial trials**

Some items in the SPIRIT (Standard Protocol Items: Recommendations for Interventional Trials) and CONSORT (Consolidated Standards of Reporting Trials) extension checklists might be harder to report, or could require adaptation for factorial trials that deviate from the standard 2×2 design.

As the number of factors being evaluated increases, so too does the chance that some treatments interact. For instance, in a 2×2×2 trial with factors A, B, and C, treatment A can interact with either treatment B or treatment C. Thus, investigators must assess and report these interactions. However, evaluating multiple interactions increases the risk of false-positive findings, which can affect interpretation.

Reporting of patient flow and outcome data by treatment group helps readers understand the impact of potential interactions on results. However, reporting such data might be challenging when the number of factors is large. For instance, a 2^6^ trial, with six factors, would have 64 distinct treatment groups; it might not be feasible or meaningful to report results for each of the 64 groups separately. Some groups might therefore need to be combined for reporting; however, combining groups in this way might also affect interpretation of results.

For trials that recruit a different number of participants for each factor (eg, due to a partial factorial design or the early stopping of some factors), results for each factor might become available at different times, which can lead to challenges in reporting key items. If factor A is complete, but recruitment to factor B is still ongoing, transparent reporting for factor A requires information on its interaction with treatment B and outcome data across treatment groups A, groups A and B, and so on. However, reporting results in this way could unblind results for the ongoing factor B comparison, affecting its integrity. Therefore, there is some trade-off between transparent reporting of available results and maintaining the integrity of ongoing comparisons.

In general, more complex factorial designs are often harder for readers to understand and to quantify the effects of potential interactions or other challenges on the trial results. This is not in itself a reason to avoid more complex designs, as such designs often bring other benefits. However, it makes transparent reporting of such trials even more essential, and requires special consideration on how key issues can be reported in the face of added complexity.

## SPIRIT and CONSORT checklists for factorial trials

The CONSORT and SPIRIT checklists for factorial trials were developed concurrently, and the methods have been described elsewhere.^48^ ^49^ Table 1 and table 2 show the standard CONSORT and SPIRIT checklists together with suggested modifications for factorial trials. In this section, we discuss each extension checklist item, explain the rationale, and provide examples of good reporting.

**Table 1:** Checklist for reporting of factorial randomised trials: extension of the CONSORT 2010 statement*†

Section/topic	Item No	Checklist item from CONSORT 2010 statement	Extension for factorial trials
**Title and abstract**			
Title	1a	Identification as a randomised trial in the title	Identification as a factorial randomised trial in the title
Abstract	1b	Structured summary of trial design, methods, results, and conclusions (for specific guidance, see CONSORT for abstracts)	See separate factorial checklist for abstracts
**Introduction**			
Background	2a	Scientific background and explanation of rationale	Scientific background and rationale for using a factorial design, including whether an interaction is hypothesised
Objectives	2b	Specific objectives or hypotheses	Specific objectives or hypotheses and a statement of which treatment groups form the main comparisons†
**Methods**			
Trial design	3a	Description of trial design (such as parallel, factorial) including allocation ratio	Description of the type of factorial trial (such as full or partial, number of factors, levels within each factor†) and allocation ratio
Change from protocol	3b	Important changes to methods after trial commencement (such as eligibility criteria), with reasons	—
Participants	4a	Eligibility criteria for participants	Eligibility criteria for each factor, noting any differences, if applicable
Setting and location	4b	Settings and locations where the data were collected	—
Interventions	5	The interventions for each group with sufficient details to allow replication, including how and when they were actually administered	—
Outcomes	6a	Completely defined prespecified primary and secondary outcome measures, including how and when they were assessed	—
Changes to outcomes	6b	Any changes to trial outcomes after the trial commenced, with reasons	—
Sample size	7a	How sample size was determined	How sample size was determined for each main comparison, including whether an interaction was assumed in the calculation
Interim analyses and stopping guidelines	7b	When applicable, explanation of any interim analyses and stopping guidelines	When applicable, explanation of any interim analyses and stopping guidelines, noting any differences across main comparisons and reasons for differences
Randomisation			
Sequence generation	8a	Method used to generate the random allocation sequence	—
	8b	Type of randomisation; details of any restriction (such as blocking and block size)	Type of randomisation; details of any restriction (such as blocking and block size), and, if applicable, whether participants were randomised to factors at different time points
Allocation concealment mechanism	9	Mechanism used to implement the random allocation sequence (such as sequentially numbered containers), describing any steps taken to conceal the sequence until interventions were assigned	—
Implementation	10	Who generated the random allocation sequence, who enrolled participants, and who assigned participants to interventions	—
Blinding	11a	If done, who was blinded after assignment to interventions (for example, participants, care providers, those assessing outcomes)	—
Similarity of interventions	11b	If relevant, description of the similarity of interventions	—
Statistical methods	12a	Statistical methods used to compare groups for primary and secondary outcomes	Statistical methods used for each main comparison for primary and secondary outcomes, including: • Whether the target treatment effect for each main comparison pertains to the effect in the presence or absence of other factors • Approach to analysis, such as factorial or multiarm • How the approach was chosen, such as prespecified or based on estimated interaction • If factorial approach used, whether factors were adjusted for each other • If applicable, how non-concurrent recruitment to factors was handled • Method(s) used to evaluate statistical interaction(s)
Additional analyses	12b	Methods for additional analyses, such as subgroup analyses and adjusted analyses	—
**Results**			
Participant flow (a diagram is strongly recommended)	13a	For each group, the numbers of participants who were randomly assigned, received intended treatment, and were analysed for the primary outcome	For each main comparison, the number of participants who were randomly assigned, received intended treatment, and were analysed for the primary outcome
Losses and exclusions	13b	For each group, losses and exclusions after randomisation, together with reasons	For each main comparison, losses and exclusions after randomisation, together with reasons
Recruitment	14a	Dates defining the periods of recruitment and follow-up	Dates defining the periods of recruitment and follow-up for each factor, noting any differences, with reasons
Trial end	14b	Why the trial ended or was stopped	—
Baseline data	15	A table showing baseline demographic and clinical characteristics for each group	A table showing baseline demographic and clinical characteristics for each main comparison
Numbers analysed	16	For each group, number of participants (denominator) included in each analysis and whether the analysis was by original assigned groups	For each main comparison, the number of participants (denominator) included in each analysis and whether the analysis was by original assigned groups
Outcomes and estimation	17a	For each primary and secondary outcome, results for each group, and the estimated effect size and its precision (such as 95% confidence interval)	For each primary and secondary outcome, results for each main comparison, the estimated effect size, and its precision (such as 95% confidence interval); for each primary outcome, the estimated interaction effect and its precision; and if done, the estimated interaction effects and precision for secondary outcomes
Binary outcomes	17b	For binary outcomes, presentation of both absolute and relative effect sizes is recommended	—
Ancillary analyses	18a	Results of any other analyses performed, including subgroup analyses and adjusted analyses, distinguishing prespecified from exploratory	—
Additional data summaries‡	18b	—	Participant flow, losses and exclusions, baseline data, and outcome data (including primary and secondary outcomes, harms, and adherence) presented by treatment groups†
Harms	19	All important harms or unintended effects in each group (for specific guidance see CONSORT for harms)	All important harms or unintended effects for each main comparison
**Discussion**			
Limitations	20	Trial limitations, addressing sources of potential bias, imprecision, and, if relevant, multiplicity of analyses	—
Generalisability	21	Generalisability (external validity, applicability) of the trial findings	—
Interpretation	22	Interpretation consistent with results, balancing benefits and harms, and considering other relevant evidence	—
**Other information**			
Registration	23	Registration number and name of trial registry	—
Protocol	24	Where the full trial protocol can be accessed, if available	—
Funding	25	Sources of funding and other support (such as supply of drugs), role of funders	—

CONSORT=consolidated standards of reporting trials.

* This checklist should be read in conjunction with the CONSORT 2010 checklist https://www.equator-network.org/reporting-guidelines/consort/ and statement explanation and elaboration paper^3^ for important clarification on the items. The CONSORT-factorial checklist is licensed by the CONSORT-factorial Group under the Creative Commons Attribution-NonCommercial-NoDerivs 4.0 International license.

† Each overall intervention group to be compared is a factor (eg, in a 2×2 trial with factors A and B, active A and control A together comprise one factor and active B and control B together comprise another factor). The specific interventions within a factor are the levels (eg, active A and control A are the two levels of factor A). The unique combinations of factors and levels are treatment groups (eg, a 2×2 trial with factors A and B will have four treatment groups: active A plus control B, active A plus active B, and so on). What treatment groups compared against each other to draw main conclusions about the effectiveness of each intervention is the main comparison.

‡ New item.

**Table 2:** Checklist for reporting of factorial, parallel group randomised trials: extension of the SPIRIT 2013 statement*†

Section/topic	Item No	Checklist item from SPIRIT 2013	Extension for factorial trials
**Administrative information**			
Title	1	Descriptive title identifying the study design, population, interventions, and, if applicable, trial acronym	Descriptive title identifying the study as a factorial randomised trial, as well as the population, interventions, and, if applicable, trial acronym
Trial registration	2a	Trial identifier and registry name. If not yet registered, name of intended registry	—
	2b	All items from the World Health Organization Trial Registration Data Set	—
Protocol version	3	Date and version identifier	—
Funding	4	Sources and types of financial, material, and other support	—
Roles and responsibilities	5a	Names, affiliations, and roles of protocol contributors	—
	5b	Name and contact information for the trial sponsor	—
	5c	Role of study sponsor and funders, if any, in study design; collection, management, analysis, and interpretation of data; writing of the report; and the decision to submit the report for publication, including whether they will have ultimate authority over any of these activities	—
	5d	Composition, roles, and responsibilities of the coordinating centre, steering committee, endpoint adjudication committee, data management team, and other individuals or groups overseeing the trial, if applicable (see item 21a for data monitoring committee)	—
**Introduction**			
Background and rationale	6a	Description of research question and justification for undertaking the trial, including summary of relevant studies (published and unpublished) examining benefits and harms for each intervention	Description of research question and justification for undertaking the trial, including summary of relevant studies (published and unpublished) examining benefits and harms for each intervention, and rationale for using a factorial design, including whether an interaction is hypothesised
	6b	Explanation for choice of comparators	—
Objectives	7	Specific objectives or hypotheses	Specific objectives or hypotheses and a statement of which treatment groups form the main comparisons†
Trial design	8	Description of trial design including type of trial (eg, parallel group, crossover, factorial, single group), allocation ratio, and framework (eg, superiority, equivalence, non-inferiority, exploratory)	Description of the type of factorial trial (eg, full or partial, number of factors, levels within each factor), allocation ratio, and framework (eg, superiority, equivalence, non-inferiority, exploratory)
**Methods: Participants, interventions, and outcomes**			
Study setting	9	Description of study settings (eg, community clinic, academic hospital) and list of countries where data will be collected. Reference to where list of study sites can be obtained	—
Eligibility criteria	10	Inclusion and exclusion criteria for participants. If applicable, eligibility criteria for study centres and individuals who will perform the interventions (eg, surgeons, psychotherapists)	Inclusion and exclusion criteria for each factor, noting any differences if applicable. If applicable, eligibility criteria for study centres and individuals who will perform the interventions (eg, surgeons, psychotherapists)
Interventions	11a	Interventions for each group with sufficient detail to allow replication, including how and when they will be administered	—
	11b	Criteria for discontinuing or modifying allocated interventions for a given trial participant (eg, drug dose change in response to harms, participant request, or improving/worsening disease)	—
	11c	Strategies to improve adherence to intervention protocols, and any procedures for monitoring adherence (eg, drug tablet return, laboratory tests)	—
	11d	Relevant concomitant care and interventions that are permitted or prohibited during the trial	—
Outcomes	12	Primary, secondary, and other outcomes, including the specific measurement variable (eg, systolic blood pressure), analysis metric (eg, change from baseline, final value, time to event), method of aggregation (eg, median, proportion), and time point for each outcome. Explanation of the clinical relevance of chosen efficacy and harm outcomes is strongly recommended	—
Participant timeline	13	Time schedule of enrolment, interventions (including any run-ins and washouts), assessments, and visits for participants. A schematic diagram is highly recommended	—
Sample size	14	Estimated number of participants needed to achieve study objectives and how it was determined, including clinical and statistical assumptions supporting any sample size calculations	Estimated number of participants needed to achieve study objectives and how it was determined for each main comparison, including clinical and statistical assumptions supporting any sample size calculations, such as whether an interaction was assumed in the calculation
Recruitment	15	Strategies for achieving adequate participant enrolment to reach target sample size	—
**Methods: Assignment of interventions (for controlled trials)**			
Allocation			
Sequence generation	16a	Method of generating the allocation sequence (eg, computer generated random numbers), and list of any factors for stratification. To reduce predictability of a random sequence, details of any planned restriction (eg, blocking) should be provided in a separate document that is unavailable to those who enrol participants or assign interventions	Method of generating the allocation sequence (eg, computer generated random numbers), list of any factors for stratification, and whether participants were allocated to factors at different time points, if applicable. To reduce predictability of a random sequence, details of any planned restriction (eg, blocking) should be provided in a separate document that is unavailable to those who enrol participants or assign interventions
Allocation concealment mechanism	16b	Mechanism of implementing the allocation sequence (eg, central telephone; sequentially numbered, opaque, sealed envelopes), describing any steps to conceal the sequence until interventions are assigned	—
Implementation	16c	Who will generate the allocation sequence, who will enrol participants, and who will assign participants to interventions	—
Blinding (masking)	17a	Who will be blinded after assignment to interventions (eg, trial participants, care providers, outcome assessors, data analysts), and how	—
	17b	If blinded, circumstances under which unblinding is permissible, and procedure for revealing a participant’s allocated intervention during the trial	—
**Methods: Data collection, management, and analysis**			
Data collection methods	18a	Plans for assessment and collection of outcome, baseline, and other trial data, including any related processes to promote data quality (eg, duplicate measurements, training of assessors) and a description of study instruments (eg, questionnaires, laboratory tests) along with their reliability and validity, if known. Reference to where data collection forms can be found, if not in the protocol	—
	18b	Plans to promote participant retention and complete follow-up, including list of any outcome data to be collected for participants who discontinue or deviate from intervention protocols	—
Data management	19	Plans for data entry, coding, security, and storage, including any related processes to promote data quality (eg, double data entry; range checks for data values). Reference to where details of data management procedures can be found, if not in the protocol	—
Statistical methods	20a	Statistical methods for analysing primary and secondary outcomes. Reference to where other details of the statistical analysis plan can be found, if not in the protocol	Statistical methods for each main comparison for primary and secondary outcomes, including: • Whether the target treatment effect for each main comparison pertains to the effect in the presence or absence of other factors • Approach, such as factorial or multiarm • How the approach will be chosen, such as prespecified or based on estimated interaction • If factorial approach to analysis will be used, whether factors will be adjusted for each other • Method(s) for evaluating statistical interactions, and which outcomes (in addition to the primary) they will be applied to • If applicable, how nonconcurrent recruitment to factors will be handled • Reference to where other details of the statistical analysis plan can be found, if not in the protocol
	20b	Methods for any additional analyses (eg, subgroup and adjusted analyses)	—
	20c	Definition of analysis population relating to protocol non-adherence (eg, as randomised analysis), and any statistical methods to handle missing data (eg, multiple imputation)	—
**Methods: Monitoring**			
Data monitoring	21a	Composition of data monitoring committee (DMC); summary of its role and reporting structure; statement of whether it is independent from the sponsor and competing interests; and reference to where further details about its charter can be found, if not in the protocol. Alternatively, an explanation of why a DMC is not needed	—
	21b	Description of any interim analyses and stopping guidelines, including who will have access to these interim results and make the final decision to terminate the trial	Description of any interim analyses and stopping guidelines, noting any differences across main comparisons, with reasons, and who will have access to these interim results and make the final decision to terminate the trial
Harms	22	Plans for collecting, assessing, reporting, and managing solicited and spontaneously reported adverse events and other unintended effects of trial interventions or trial conduct	—
Auditing	23	Frequency and procedures for auditing trial conduct, if any, and whether the process will be independent from investigators and the sponsor	—
**Ethics and dissemination**			
Research ethics approval	24	Plans for seeking research ethics committee/institutional review board (REC/IRB) approval	—
Protocol amendments	25	Plans for communicating important protocol modifications (eg, changes to eligibility criteria, outcomes, analyses) to relevant parties (eg, investigators, REC/IRBs, trial participants, trial registries, journals, regulators)	—
Consent or assent	26a	Who will obtain informed consent or assent from potential trial participants or authorised surrogates, and how (see item 32)	—
	26b	Additional consent provisions for collection and use of participant data and biological specimens in ancillary studies, if applicable	—
Confidentiality	27	How personal information about potential and enrolled participants will be collected, shared, and maintained in order to protect confidentiality before, during, and after the trial	—
Declaration of interests	28	Financial and other competing interests for principal investigators for the overall trial and each study site	—
Access to data	29	Statement of who will have access to the final trial dataset, and disclosure of contractual agreements that limit such access for investigators	—
Ancillary and post-trial care	30	Provisions, if any, for ancillary and post-trial care, and for compensation to those who suffer harm from trial participation	—
Dissemination policy	31a	Plans for investigators and sponsor to communicate trial results to participants, healthcare professionals, the public, and other relevant groups (eg, via publication, reporting in results databases, or other data sharing arrangements), including any publication restrictions	—
	31b	Authorship eligibility guidelines and any intended use of professional writers	—
	31c	Plans, if any, for granting public access to the full protocol, participant level dataset, and statistical code	—
**Appendices**			
Informed consent materials	32	Model consent form and other related documentation given to participants and authorised surrogates	—
Biological specimens	33	Plans for collection, laboratory evaluation, and storage of biological specimens for genetic or molecular analysis in the current trial and for future use in ancillary studies, if applicable	—

SPIRIT=standard protocol items: recommendations for interventional trials.

* It is strongly recommended that this checklist is read in conjunction with the SPIRIT 2013 statement^1^ for important clarification on the items.

† Each overall intervention group to be compared is a factor (eg, in a 2×2 trial with factors A and B, active A and control A together comprise one factor, and active B and control B together comprise another factor). The specific interventions within a factor are the levels (eg, active A and control A are the two levels of factor A). The unique combinations of factors and levels are treatment groups (eg, in a 2×2 trial with factors A and B, there will be four treatment groups: active A plus control B, active A plus active B, etc). What treatment groups will be compared against each other to draw main conclusions about the effectiveness of each intervention is the main comparison.

We have grouped similar items from the CONSORT and SPIRIT checklists together (eg, CONSORT item 1a and SPIRIT item 1, which both relate to the title), and listed the factorial specific extension below the original items (full items incorporating the factorial specific extension are available in table 1 and table 2). We provide a single explanation that applies to both CONSORT and SPIRIT items. The examples of good reporting have been chosen on the basis of how well they reported the factorial specific extension of the item, which is listed below the original items.

## Title

- CONSORT 2010 item 1a: Identification as a randomised trial in the title
- SPIRIT 2013 item 1: Descriptive title identifying the study design, population, interventions, and, if applicable, trial acronym
- Extension for factorial trials: Identification as a factorial randomised trial in the title

SPIRIT example: “Peripherally InSerted CEntral catheter dressing and securement in patients with cancer: the PISCES trial. Protocol for a 2×2 factorial, superiority randomised controlled trial”.^50^

CONSORT example: “Progressive exercise compared with best practice advice, with or without corticosteroid injection, for the treatment of patients with rotator cuff disorders (GRASP): a multicentre, pragmatic, 2×2 factorial, randomised controlled trial”.^51^

### Explanation

Including this key design feature in the title facilitates rapid identification of article relevance when searching electronic databases. Further, by clearly signalling the factorial design, readers can consider implications for the design and analysis methods, as well as any potential limitations.^8^ ^21^ ^34^ ^39^ ^40^ ^41^

## Rationale

- CONSORT 2010 item 2a: Scientific background and explanation of rationale
- SPIRIT 2013 item 6a: Description of research question and justification for undertaking the trial, including summary of relevant studies (published and unpublished) examining benefits and harms for each intervention
- Extension for factorial trials: Rationale for using a factorial design, including whether an interaction is hypothesised

SPIRIT example: “We have chosen a factorial design because it is an efficient way to evaluate more than one intervention in a single trial . . . However, the size benefits gains of this design only occur when the assumption of no interaction between the two interventions is met. We do not anticipate any interaction effects.”^52^

CONSORT example 1: “[NeoCLEAR] was designed to establish the optimal lumbar puncture technique in newborn infants in terms of the effects of infant position (sitting vs lying) and timing of stylet removal (early vs late) on lumbar puncture success . . . An efficient 2×2 factorial design was used to allow simultaneous evaluation of the two techniques, predicated on no plausible reason to expect an interaction - ie, a differential effect of one intervention dependent on the presence or absence of the other.”^53^

CONSORT example 2: “To evaluate the interaction between type of intervention delivered (decision aid v. control pamphlet) and mode of delivery (by clinician during visit v. by clinician-researcher before the visit), we used a 2×2 factorial design.”^54^

### Explanation

Factorial designs can be used for different reasons (box 2),^19^ ^20^ ^21^ ^22^ such as evaluating the effect of more than one intervention in a single trial (2-in-1 trials),^33^ ^34^ ^39^ ^42^ ^43^ evaluating whether an interaction between treatments exists, or identifying the best overall combination of factors.^31^ These aims are generally mutually exclusive, as each one requires different methodology, including different sample size calculations and analysis strategies. A clear statement of the rationale for why the factorial design was chosen helps orientate readers towards the key objectives and alerts them to the assumptions and methodological features required for the trial to satisfactorily meet those objectives.

Trials in which the factorial design has been chosen for efficiency (2-in-1 trials) rely on the assumption that treatments work independently, meaning that the effect of one intervention is assumed to be consistent in the presence or absence of other intervention(s). If treatments interact, then the assumptions underpinning the 2-in-1 trial motivation are no longer valid.^19^ ^20^ ^21^ ^22^ This situation can lead to biased treatment effect estimates and challenge the interpretation of the results (see factorial extension for CONSORT item 12a below).^27^ ^30^ ^33^ ^34^ ^35^ ^39^ ^55^ Therefore, understanding whether an interaction was hypothesised helps to facilitate critical appraisal of the validity of the design and methods.

## Objectives

- CONSORT 2010 item 2b: Specific objectives or hypotheses
- SPIRIT 2013 item 7: Specific objectives or hypotheses
- Extension for factorial trials: A statement of which treatment groups form the main comparisons

SPIRIT example: “The primary analysis will be a comparison of ECF [enhanced case finding intervention] Vs no ECF (arms 2 & 4 vs arms 1 & 3) and of HH [household intervention] Vs no HH (arms 3 & 4 vs arms 1 & 2).”^56^

CONSORT example: “The four allocated groups were general practitioners’ use of C reactive protein testing (1), training in enhanced communication skills (2), the interventions combined (3), and usual care (4). The groups were combined for analysis as follows: factor A, C reactive protein test (cells 1 and 3) compared with no test (2 and 4) . . . and factor B, training in enhanced communication skills (2 and 3) compared with no training (1 and 4)”.^57^

### Explanation

Factorial trials allow investigators to estimate the effect of an intervention in different ways. For instance, in a 2×2 factorial trial with factors A and B, participants are assigned to one of four treatment groups (A alone, B alone, both A and B, and double control (not A and not B)). Then, for an assessment of factor A, investigators could compare all A versus all not A; A alone versus double control; and A+B versus B alone. The number of potential comparisons multiplies rapidly as the number of treatment groups increases. A statement of which treatment groups form these comparisons increases clarity and facilitates interpretation of results.^8^

## Trial design

- CONSORT 2010 item 3a: Description of trial design (such as parallel, factorial) including allocation ratio
- SPIRIT 2013 item 8: Description of trial design including type of trial (eg, parallel group, crossover, factorial, single group), allocation ratio, and framework (eg, superiority, equivalence, non-inferiority, exploratory)
- Extension for factorial trials: Description of the type of factorial trial (such as a full or partial, number of factors and levels within each factor)

SPIRIT example: “POISE-3 is an international RCT [randomised controlled trial] of 10,000 adults at risk of bleeding and cardiovascular complications who are undergoing noncardiac surgery. Patients are randomized to receive intraoperative TXA [tranexamic acid] or placebo. Using a 2×2 partial factorial design, patients taking ≥1 antihypertensive medication are also randomized to a hypotension-avoidance or a hypertension-avoidance strategy.”^58^

CONSORT example: “NeoCLEAR was an open-label, 2×2 full factorial, randomised, controlled trial with an internal pilot . . . [It] was designed to establish the optimal lumbar puncture technique in newborn infants in terms of the effects of infant position (sitting vs lying) and timing of stylet removal (early vs late) on lumbar puncture success”.^53^

### Explanation

Providing specific details relating to the type and structure of the factorial design allows assessment of the potential for bias, and whether the sample size and analysis methods are appropriate. This detail includes reporting the number of factors being evaluated and the levels within each factor^8^ ^41^; for example, “2×2” is often used to signify two factors, each with two levels.

Most trials use a full factorial design, whereby all factors and factor levels are combined, and each participant is eligible to be allocated to each combination of factors.^23^ ^24^ ^29^ Other factorial designs can be fractional or partial. In a fractional factorial design, certain combinations of factors are not used for any participants, for example, if participants are randomised between only six of the eight possible combinations in a 2×2×2 design. In a partial factorial design, some participants are only eligible to be randomised for certain factors.^23^ ^29^

## Eligibility criteria

- CONSORT 2010 item 4a: Eligibility criteria for participants
- SPIRIT 2013 item 10: Inclusion and exclusion criteria for participants. If applicable, eligibility criteria for study centres and individuals who will perform the interventions (eg, surgeons, psychotherapists)
- Extension for factorial trials: Eligibility criteria for each factor, noting any differences, if applicable

SPIRIT example: “The broad inclusion criteria are used for both [factors], whereby adult patients who have received ECMO [extracorporeal membrane oxygenation] for any reason within 24 h and 7 days . . . are eligible . . . As ECMO is often initiated by a specialist team to rescue and transfer patients to larger hospitals in China, a timeframe of 24 h after ECMO implantation was used for early CRRT [continuous renal replacement therapy] in [factor A], while a relatively stable hemodynamic status (dopamine/dobutamine < 5 μg/kg/min, with no administration of adrenaline or norepinephrine) and within 7 days after initiation of ECMO was required for eligibility into [factor B].”^59^

CONSORT example: “Patients were eligible for the trial if they were admitted to hospital with a clinical stroke syndrome due to ischaemic or haemorrhagic stroke, were aged 18 years or older, had a motor deficit in their arm or leg or both, had a systolic blood pressure of 140–220 mm Hg, and could be treated within 48 h of onset . . . [Eligible] patients were randomly assigned to 7 days of treatment with transdermal glyceryl trinitrate . . . or no glyceryl trinitrate. In addition, a subset of patients who were taking blood pressure-lowering drugs immediately before their stroke were randomly assigned to continue or stop these drugs temporarily in a partial-factorial design.”^60^

### Explanation

A full description of the eligibility criteria is required to help readers determine the population to whom the results of the trial apply. A description of the differences across factors (if applicable), along with reasons, alerts readers that trial results could apply to different populations. Further, trials in which eligibility differs across factors (eg, partial factorial trials) have specific methodological considerations that can lead to biases if not handled properly (see factorial extension for CONSORT item 3a above).

## Sample size

- CONSORT 2010 item 7a: How sample size was determined
- SPIRIT 2013 item 14: Estimated number of participants needed to achieve study objectives and how it was determined, including clinical and statistical assumptions supporting any sample size calculations
- Extension for factorial trials: How sample size was determined for each main comparison, including whether an interaction was assumed in the calculation

SPIRIT example: “This pragmatic, multicentre, 2×2 factorial, superiority RCT [randomised controlled trial] will test the clinical efficacy and cost-effectiveness of (1) dressings (CHG [chlorhexidine gluconate] disc vs no disc) and (2) securements (SED [securement device] vs ISD [integrated securement dressing]) . . . [The hypothesis for the dressings comparisons is the] use of a CHG disc will reduce the incidence of PICC CABSI [peripherally inserted central catheters catheter-associated bloodstream infection] compared with the use of no disc, [and the hypothesis for the securements comparisons is the] use of an ISD will reduce the incidence of composite PICC failure, compared with the use of SED. [For our dressings hypothesis,] a one-sided inequality test of two proportions calculated that 602 PICCs per group would detect reduced CABSI incidence from 8% to 4% with 90% power (p=0.05). [For our securements hypothesis,] our local baseline PICC failure is 26% with SED [and] we hypothesise 19% failure in the combined ISD groups . . . A one-sided inequality test of two proportions calculated that 608 PICCs per group (608 ISD; 608 SED) [are required] for 90% power (p=0.05). Because of the factorial design, we used the comparison that required the larger sample (608 per group), plus 2% for potential attrition, thus 620 per group (total trial 1240). We assumed no interaction effect between the interventions.”^50^

CONSORT example: “The target sample size was 704 participants, based on 90% power to detect a minimally clinically important between-group difference of 8 points on the SPADI [shoulder pain and disability index score] total scale, assuming a baseline SD [standard deviation] of 24.3. This difference is equivalent to a standardised effect size of 0.33, which required a sample size of 550 participants . . . Allowing for a potential loss to follow-up at 12 months of 20% increased the sample size to 688 participants. We further increased the sample size to 704 participants to take into account the potential for a small clustering effect by physiotherapist (interclass correlation 0.001). The sample size assumed no interaction effect and was powered for the two main-effect comparisons: (1) progressive exercise versus best practice advice to investigate the effects of progressive exercise and (2) subacromial corticosteroid injection versus no injection to investigate the effects of corticosteroid injection.”^51^

### Explanation

The sample size calculation should be guided by the main trial objectives, and thus will differ depending on whether the aim is to evaluate multiple interventions in a single trial, or to detect an interaction.

Interactions affect the required sample size.^31^ ^34^ ^40^ ^42^ A statement relating to the assumptions regarding the presence or absence of interaction in the calculation will assist readers in understanding how the calculation was performed, and whether it is appropriate for the trial objectives.

For 2-in-1 factorial trials, a sample size calculation is typically performed for each main comparison using the same approach as for a two-arm parallel group trial (ie, using the same desired level of power, significance level, etc); however, the required number of participants per group is then multiplied by the number of levels within the comparison to obtain the overall sample size, rather than by the number of treatment groups in the trial (eg, in a 2×2 trial, this number is multiplied by two levels, and not by four treatment groups).

Then, once the sample size has been calculated for each main comparison, the largest of these is chosen if they differ across comparisons. A difference might occur if different interventions are expected to produce different treatment effects, if different primary outcomes are used for each factor, or if the number of levels differs between factors.^42^ ^61^

Sample size calculations based on a binary primary outcome might require special consideration to account for the assumed effect of each other intervention on the event rate in the control arm.^62^ For example, in a 2×2 factorial design, assume that (1) the event rate in the double control group is 40%; and that (2) both treatments reduce the absolute event rate by 5 percentage points, or an equivalent relative reduction of 12.5%. For the comparison between all A and all not A, the assumed event rate in the all-not-A group is thus 37.5%, because half of these participants also receive treatment B. This difference from the event rate in the double control group might require a reduction or increase in sample size, depending on whether the same absolute or relative effect is to be detected.

## Interim analyses

- CONSORT 2010 item 7b: When applicable, explanation of any interim analyses and stopping guidelines
- SPIRIT 2013 item 21b: Description of any interim analyses and stopping guidelines, including who will have access to these interim results and make the final decision to terminate the trial
- Extension for factorial trials: When applicable, explanation of any interim analyses and stopping guidelines, noting any differences across main comparisons and reasons for differences

SPIRIT example: “We will conduct an interim analysis to monitor the treatment benefits. The interim analysis will be performed when two-thirds of the entire patient follow-up is completed (i.e. 1520 person-years). At this point, 91.7% (1886) patients will have been recruited into the trial. We use the O’Brien-Fleming Method to calculate the stopping boundary. We will maintain the overall specified type I error rate of 0.05 for the comparison of soap solution versus normal saline, and the threshold 2-sided significance level is 0.012 for the interim analysis. We will maintain the overall specified type I error of 0.0188 for each of the three pairwise comparisons of irrigation solutions, and the threshold 2-sided significance level is 0.003.”^63^

CONSORT example: “Annual interim analyses were planned after 30% of the expected number of failures had occurred . . . a test of futility was to be conducted at each interim analysis for each factor. If the 99.5% confidence limit for the HR [hazard ratio] excluded the alternate hypothesis (HR, 0.82), then the trial would not be able to be positive and a recommendation would be made to the DSMC [data safety and monitoring committee] to discontinue random assignment to that factor.

“At the time of the first interim analysis in September 2010, the observed HR for DFS [disease-free survival] for doxorubicin-cyclophosphamide with filgrastim versus doxorubicin-cyclophosphamide once every 2 weeks was 1.21 (adjusting for the paclitaxel randomisation). The 99.5% CI [confidence interval] was 0.90 to 1.64, suggesting that it would be futile to continue randomisation to this factor. On the basis of the recommendation of the DSMC, accrual to the trial was suspended in November 2010. The trial reopened in December 2010 with all patients assigned to four cycles of doxorubicin-cyclophosphamide administered once every 2 weeks and randomly assigned only to the paclitaxel factor. At the third interim analysis in September 2012, the futility boundary for the comparison of the two paclitaxel schedules was crossed, with a Cox model adjusting for the doxorubicin-cyclophosphamide arms producing an HR of 1.08 (99.5% CI, 0.83 to 1.39), thus excluding 0.82. On the basis of this analysis, the DSMC recommended releasing the results.”^64^

### Explanation

Many trials perform interim analyses to assess whether the trial should be stopped early, either due to benefit, harm, or futility.^8^ In factorial trials, the plan for interim analyses and subsequent stopping guidelines might be different for each factor.^46^ For example, one factor could be drug related and another not related to drugs; here, safety monitoring might be more important in the first factor than the second. Conversely, different factors might contain different numbers of levels, which could affect the specific stopping thresholds for each.

Early stopping of one factor but not the others can lead to non-concurrent enrolment of participants (eg, participants who are randomised for factor A, but not factor B), which has implications for the analysis population (see factorial extension for CONSORT item 12a below).^31^ ^46^

## Randomisation

- CONSORT 2010 item 8b: Type of randomisation; details of any restriction (such as blocking and block size)
- SPIRIT 2013 item 16a: Method of generating the allocation sequence (eg, computer generated random numbers) and list of any factors for stratification. To reduce predictability of a random sequence, details of any planned restriction (eg, blocking) should be provided in a separate document that is unavailable to those who enrol participants or assign interventions
- Extension for factorial trials: If applicable, whether participants were allocated to factors at different time points

SPIRIT example: “We [will randomise] patients 1:1 to dabigatran or placebo and, using a partial factorial design, 1:1 to omeprazole or placebo. Both randomizations [will occur] at the same time.”^65^

CONSORT example: “This was a superiority trial with the two groups in Protocol 1 allocated 1:1 and stratified by study site. Allocation for Protocol 2 occurred 30 min after the drug infusion for patients who had not converted and was stratified by site and Protocol 1 allocation.”^66^

### Explanation

Participants are sometimes randomised for factors at different time points. For example, they might be allocated to factor A versus not A at diagnosis of disease, then again to factor B versus not B at a later time point, once treatment A is complete and the response is evaluated. The time point of randomisation for each factor might inform key design features, such as the baseline period, duration of follow-up, and likelihood of treatments interacting; therefore, if the timing of randomisation for factors is different, this should be reported.^34^

## Statistical methods

- CONSORT 2010 item 12a: Statistical methods used to compare groups for primary and secondary outcomes
- SPIRIT 2013 item 20a: Statistical methods for analysing primary and secondary outcomes. Reference to where other details of the statistical analysis plan can be found, if not in the protocol
- Extension for factorial trials: Statistical methods used for each main comparison for primary and secondary outcomes, including whether the target treatment effect for each main comparison pertains to the effect in the presence or absence of other factors

SPIRIT example: “In the first instance, the objective is to estimate the effect of progressive exercise vs. best practice advice without corticosteroid injection (that is, we are interested in estimating the effect that would be observed in a two-arm parallel group trial where standard of care was no injection). The same objective applied for the corticosteroid comparison, i.e. to estimate the effect of corticosteroid injection vs. no injection without progressive exercise.” (Adapted from Hopewell and colleagues.^67^)

CONSORT example: “For the EPA [eicosapentaenoic acid] comparison, the objective was to evaluate the effect of EPA alone vs. placebo alone, i.e. both without the use of aspirin. Similarly, for the aspirin comparison, the objective was to evaluate the effect of aspirin alone vs. placebo alone, i.e. both without the use of EPA.” (Adapted from Hull and colleagues.^16^)

We were not able to find examples of good reporting tackling the item required. Therefore, in the examples above, we adapted a published protocol and a published trial report to illustrate examples of good reporting.

### Explanation

Although it is essential to provide an accurate description of the trial design and analysis, this description is not always sufficient to allow readers to infer the exact question being addressed through the reported treatment comparisons.^68^ ^69^ ^70^ ^71^ ^72^ A particular problem in factorial trials is that the treatment groups used for comparison are not always the same as those in which we wish to estimate the treatment effect.^33^ ^48^ ^49^ For instance, 2-in-1 trials often use a factorial (or at-the-margins) analysis to compare all A with all not A for reasons of efficiency, even though interest really lies in the effect of A alone versus control (the effect of A in the absence of B). Conversely, interest might lie in the effect of A+B versus B alone (the effect of A in the presence of B), if treatment B has been demonstrated to be effective.^33^

A clear description of the target treatment effect to be estimated, including whether it pertains to the effect in the presence or absence of other factors, allows readers to understand the exact question being asked in the trial.^33^ ^68^ ^69^ ^73^ The target treatment effect is called the estimand (box 6), and is specified for each comparison.^33^ ^72^ ^73^ In the CONSORT example above, the estimand shows that, regardless of how the trial is analysed, interest for each comparison lies in the effect that would have been observed had participants not been simultaneously randomised for the other factor. Therefore, the trial is set out to investigate the effect of active EPA (eicosapentaenoic acid) versus placebo EPA without the use of aspirin (and similarly for the aspirin comparison).

Box 6 **Overview of estimands in factorial trials (adapted from Kahan et al^49^)**

**Estimands for factorial trials**

- Estimands are used to describe the research question(s) a trial aims to investigate.
- In factorial trials, different types of estimands can be specified depending on the aims.
- For 2-in-1 trials, estimands are typically based around the comparison of treatment A versus not A (and similarly for other factors). However, this estimand can be defined in different ways; for instance, it could be based on the comparison of treatment A versus not A if no one received treatment B, or as the effect of A versus not A if everyone received treatment B.
- Alternatively, the estimand for treatment A could be defined on the basis of the comparison of A versus not A, averaged across those individuals who do and those who do not receive treatment B*. However, this estimand does not typically reflect how treatments are used in practice, and so other estimands are usually more relevant for 2-in-1 trials.
- For trials aiming to determine whether treatments interact, the estimand might be based around the difference in the effects of treatment A if no one received treatment B versus if everyone received treatment B.

**Implications for statistical analysis**

- The method of statistical analysis should be chosen based on the estimand.
- For 2-in-1 trials, a factorial (at-the-margins) analysis is typically used, owing to its efficiency. However, this analysis averages across the two stratums of those individuals allocated to receive and not receive B, and so it estimates the effect of treatment A if no one receives B only if treatments A and B do not interact. If treatments do interact, it estimates an average effect of A across the strata of B, which is not usually of primary interest.
- A multiarm (inside-the-table) analysis can also estimate the effect of treatment A if no one receives B, even when treatments A and B do interact. However, because it is less efficient than the factorial analysis, it is less frequently used for 2-in-1 trials.

*This average could correspond either to some proportions defined by investigators, or to the study proportions allocated to B and not B. Therefore, the exact method of averaging should be made explicit. If this average is defined based on the study proportions, it should be clarified whether this average is based on the initially specified allocation ratio (eg, 1:1), or the final observed proportions in each stratum. These proportions could differ substantially if, for instance, randomisation to factor B is stopped partway through the trial for safety reason.

### Extension for factorial trials: Statistical methods used for each main comparison for primary and secondary outcomes, including approach to analysis, such as factorial or multiarm 

SPIRIT example: “The primary analysis for both factors (podophyllotoxin vs. imiquimod, and qHPV [quadrivalent human papillomavirus] vs. placebo vaccine) will be based on comparisons at the margins of the 2×2 table . . . meaning all participants randomised to podophyllotoxin will be compared with all participants randomised to imiquimod, and all participants randomised to qHPV vaccine will be compared with all participants randomised to saline placebo. We do not anticipate a substantial interaction between topical treatment and vaccination. However, as a secondary analysis, we will perform an interaction test between the two factors, and present results from a four-arm analysis (where each of the four treatment groups is regarded as a separate treatment arm), as is recommended for factorial trials.”^74^

CONSORT example: “The main analyses consisted of the comparison between patients allocated to aspirin versus no aspirin, and to unfractionated heparin versus no unfractionated heparin.”^75^

#### Explanation

For 2-in-1 factorial trials, a factorial (or at-the-margins) analysis is typically used, in which all participants allocated to factor A (A alone, and A+B) are compared with all those not allocated to A (B alone, and double control).^33^ ^34^ ^39^ ^42^ ^43^ This analysis takes advantage of the efficiency gains inherent in the factorial design, allowing investigators to evaluate multiple interventions in a single trial with minimal effect on the sample size. However, factorial analyses rely on the assumption that treatments do not interact and will be biased otherwise; the larger the interaction, the more extreme the bias.^30^ ^32^ ^33^ ^34^ ^35^ ^39^ ^40^ ^41^ ^42^ ^43^

An alternative approach is a multiarm (or inside-the-table) analysis, in which the trial is analysed as if a multiarm design had been used instead.^30^ ^32^ ^33^ ^34^ ^35^ ^39^ ^41^ ^42^ ^43^ In a 2×2 design, the treatment groups A alone, B alone, and A+B are each compared against double control. The multiarm analysis is unbiased even when treatments interact but is less efficient than the factorial analysis owing to the reduced sample size for each comparison, and thus will typically be underpowered.^34^ ^39^ ^42^ ^43^

For this reason, it is generally recommended that main comparisons for 2-in-1 trials are based on a factorial analysis (to take advantage of the efficiency gains inherent to the factorial design), but that multiarm analyses are routinely reported as a sensitivity analysis (see proposed framework for the analysis of 2-in-1 trials in box 7).^33^ If some evidence of an interaction exists, results from the multiarm sensitivity analyses can help identify how robust conclusions from the main factorial analyses are.

Box 7 **Framework for statistical analysis of 2-in-1 trials (adapted from Kahan et al^33^)**

1. Specify the estimand of interest for each main comparison, including whether it pertains to the effect in the presence or absence of other factors.
2. Use a factorial (at-the-margins) analysis as for the primary analysis to make use of the gain inherent from the factorial design under the assumption of no interaction.
3. Report the size of the estimated interaction and its precision (eg, using a confidence interval) to assess the plausibility of the assumption of no interaction underpinning the factorial analysis.
4. Perform a sensitivity analysis using a multiarm analysis to evaluate to what extent departures from the underlying assumption of no interaction might influence results.

A detailed description of which analytical approach is used for the main comparisons provides clarity on the assumptions underpinning the analysis, as well as any implications for interpretation.

### Extension for factorial trials: Statistical methods used for each main comparison for primary and secondary outcomes, including how the approach was chosen, such as prespecified or based on estimated interaction

SPIRIT example: “The primary statistical analysis will be carried out on the basis of intention to treat . . . There will be two main effect comparisons for this 2×2 factorial trial: (1) progressive exercise versus best practice advice and (2) subacromial corticosteroid injection versus no injection.”^67^

CONSORT example: “Analyses (factorial) were prespecified in the statistical analysis plan (appendix p18), which was approved before data lock.”^53^

#### Explanation

Because the factorial analysis is appropriate when treatments do not interact, and the multiarm analysis is appropriate when treatments do interact, investigators sometimes use a test of interaction to guide their choice of analysis strategy.^32^ ^34^ If the interaction is not statistically significant, they use a factorial analysis, and if it is significant they switch to a multiarm analysis. This approach is thought to provide a safeguard against potential interactions. However, owing to the typically low power to detect statistical interactions, as well as problems associated with choosing the method of analysis based on observed data (even when prespecified), this analysis approach can lead to biased estimates of treatment effect and incorrect type I error rates, even when treatments do not interact 32; as such, alternative approaches to choose the statistical approach are preferred (for instance, box 7). Therefore, clarifying whether the final analysis approach was prespecified or chosen based on the size of the estimated interaction increases clarity and alerts readers to the statistical implications of the analysis approach.

### Extension for factorial trials: Statistical methods used for each main comparison for primary and secondary outcomes, including method(s) used to evaluate statistical interaction(s)

SPIRIT example: “We will assess both primary outcomes for interaction. We will introduce an interaction term between the treatment indicator and the intervention indicator to test whether in (A) the effect of the metamizole treatment on the change in NRS [numeric rating scale] at day 14 is different in patients that receive the short intervention compared with the standard care group and in (B) whether the effect of the short intervention on the change in COMI [core outcome measures index] at day 42 is different in patients that receive metamizole compared with patients that receive ibuprofen.”^76^

CONSORT example: “The interaction between aspirin and mesalazine was analysed by incorporating an interaction term into the model.”^77^

#### Explanation

The presence of statistical interactions should be evaluated, either because analyses rely on the assumption that treatments do not interact, or because the interaction is itself of direct interest.^30^ ^33^ ^34^ ^39^ ^40^ ^41^ ^42^ In the simplest case of a 2×2 full factorial trial, one method is to include an interaction term between factors A and B in a statistical model. This model should not be used to estimate the effects of factors A and B directly, because the inclusion of the interaction term modifies the interpretation of other effects in the model.^33^ ^34^

### Extension for factorial trials: Statistical methods used for each main comparison for primary and secondary outcomes, including if a factorial approach used, whether factors were adjusted for each other

SPIRIT example: “The primary outcome . . . will be analysed using a logistic regression model, and will be adjusted for gender, previous occurrence of warts, HIV status and site as stratification factors, and will include both treatment factors (topical treatment and vaccination) as covariates.”^74^

CONSORT example: “Time to event endpoints were analysed by means of a Cox proportional hazards models stratified for the other randomisation (either IL [interleukin]-1 blockade [yes or no] or IL-6 blockade ([yes or no]).”^78^

#### Explanation

For factorial analyses, it can be desirable to adjust for which levels of the other factor(s) participants were allocated to. This adjustment can be done by including a term for the other factor (eg, factor B) in the statistical model,^33^ ^34^ ^42^ ^61^ similar to how baseline covariates such as age or disease stage might be adjusted for during analysis.^79^

Adjustment for other factors can increase statistical power by accounting for any variation in outcomes caused by receipt of the other factors. For certain treatment effect measures (eg, odds ratios, hazard ratios), failure to adjust for the other factors can introduce bias.^33^ Reporting whether such adjustment was undertaken alerts readers to the potential statistical implications of such an approach.

While statistical adjustment for the other factor(s) can be beneficial, as stated above, care should be taken not to adjust for the interaction between factors in a factorial analysis. Briefly, adjustment for the other factor would involve including a term for assignment for factor B (yes *v* no) in the statistical model; conversely, adjustment for the interaction involves including a term for assignment for factor B (yes *v* no), as well as the interaction term between factors A and B (which is typically derived by multiplying together factors A and B, so that the interaction term is coded as “yes” if participants were assigned to receive the active intervention for both factors).

### Extension for factorial trials: Statistical methods used for each main comparison for primary and secondary outcomes, including if applicable, how non-concurrent recruitment to factors was handled

SPIRIT example: “All patients will be randomised to one of opiate or NSAID [non-steroidal anti-inflammatory drugs] based analgesic regimen. Only patients without thoracoscopy will also be randomised to receive a large bore chest drain or a small bore chest drain . . . Only patients who do not undergo thoracoscopy will be randomised to a 24F or 12F drain, and so only non-thoracoscopy patients will be included in the primary comparison of drain sizes for each outcome.”^80^

CONSORT example: “We did a partial factorial trial of two protocols for patients with acute atrial fibrillation at 11 academic hospital emergency departments in Canada . . . Protocol 1 was a randomised . . . comparison of attempted pharmacological cardioversion with intravenous procainamide (15 mg/kg over 30 min) followed by electrical cardioversion if necessary (up to three shocks, each of ≥200 J), and placebo infusion followed by electrical cardioversion. For patients having electrical cardioversion, we used Protocol 2, a randomised . . . comparison of anteroposterior versus anterolateral pad positions. The primary analytical approach for Protocol 1 was by intention to treat . . . The comparison of the anteroposterior and anterolateral pad positions (Protocol 2) was done using a modified intention-to-treat approach, excluding patients who did not receive electrical cardioversion.”^66^

#### Explanation

Non-concurrent recruitment, in which some participants are not randomised for some factors (either because they are not eligible for that factor or because that factor has ceased recruitment due to early stopping) creates challenges for the analysis. The inclusion of such participants in the comparison for the factor to which they were not randomised can induce bias, since such an analysis does not strictly compare randomised groups (box 4).^31^ ^46^ In this case, during data analysis, only participants who are eligible to be randomised for a specific factor should be included in the comparison for that factor.^31^ ^46^ Reporting how such participants were handled during analysis (for instance, exclusion from comparisons for which they were not randomised) is necessary to understand the validity of results.

## Participants flow

- CONSORT 2010 item 13a: For each group, the numbers of participants who were randomly assigned, received intended treatment, and were analysed for the primary outcome
- Extension for factorial trials: For each main comparison, the number of participants who were randomly assigned, received intended treatment, and were analysed for the primary outcome
- CONSORT 2010 item 13b: For each group, losses and exclusions after randomisation, together with reasons
- Extension for factorial trials: For each main comparison, losses and exclusions after randomisation, together with reasons

**Fig 1:**
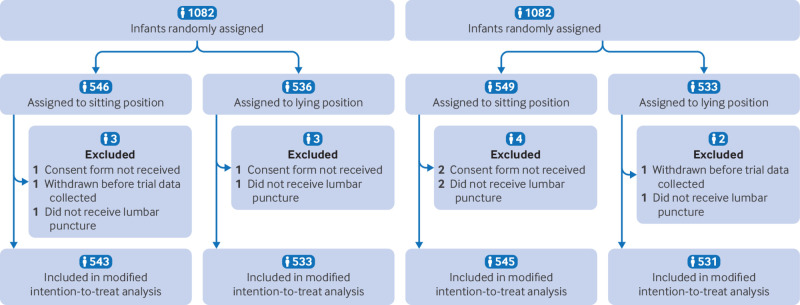
Example of a flow diagram for a factorial trial (NeoCLEAR). The NeoCLEAR trial assessed infant position and timing of stylet removal to improve lumbar puncture success in newborn babies. Figure adapted from Marshall et al.^53^ Screening and eligibility data are not shown

CONSORT example: See figure 1.^53^

### Explanation

Presenting participant flow can increase clarity and understanding. However, in factorial trials it can be difficult to understand the relevant participant flow as information relating to adherence, completeness of outcome data collection, losses to follow-up, and post-randomisation exclusion might differ across main comparisons. Presenting participant flow for each main comparison can increase clarity and understanding of results.^21^ ^30^ ^34^ ^39^ ^41^ ^42^ ^43^

## Recruitment and follow-up periods

- CONSORT 2010 item 14a: Dates defining the periods of recruitment and follow-up
- Extension for factorial trials: Dates defining the periods of recruitment and follow-up for each factor, noting any differences, with reasons

CONSORT example: “A total of 2,716 patients were randomly assigned from December 2003 to November 2010 to the original 2×2 design . . . On the basis of the recommendation of the DSMC [data safety and monitoring committee], accrual to the trial was suspended in November 2010. The trial reopened in December 2010 with all patients assigned to four cycles of doxorubicin-cyclophosphamide administered once every 2 weeks and randomly assigned only to the paclitaxel factor.”^64^

### Explanation

Different recruitment periods across factors (eg, if a factor is dropped, or a new factor is introduced), poses similar statistical issues as in a partial factorial design (see factorial extensions for CONSORT items 3a and 12a above).^46^ Describing differences in periods of recruitment across factors alerts readers to the statistical implications.

## Baseline characteristics

- CONSORT 2010 item 15: A table showing baseline demographic and clinical characteristics for each group
- Extension for factorial trials: A table showing baseline demographic and clinical characteristics for each main comparison

CONSORT example: See table 3.^53^

**Table 3:** Baseline characteristics of participants in a factorial trial (NeoCLEAR). The NeoCLEAR trial assessed infant position and timing of stylet removal to improve lumbar puncture success in newborn babies. Table adapted from Marshall et al^53^

Characteristic	Comparison 1			Comparison 2		
	Sitting (n=543)	Lying (n=533)		Early stylet removal (n=545)	Late stylet removal (n=531)	
Corrected gestational age (weeks^+days^):					
27^+0^ to 31^+6^	11 (2.0)	11 (2.1)		10 (1.8)	12 (2.3)	
32^+0^ to 36^+6^	46 (8.5)	47 (8.8)		49 (9.0)	44 (8.3)	
37^+0^ to 40^+6^	299 (55.1)	295 (55.3)		297 (54.5)	297 (55.9)	
41^+0^ to 44^+0^	187 (34.4)	180 (33.8)		189 (34.7)	178 (33.5)	
Median (IQR)	40 (39-41)	40 (39-41)		40 (39-41)	40 (39-41)	
Age (days):					
Median (IQR)	1 (1 to 2)	2 (1 to 2)		2 (1 to 2)	1 (1 to 2)	
≥3 days	70 (12.9)	70 (13.1)		74 (13.6)	66 (12.4)	
Working weight at trial entry (g):						
Median (IQR)	3500 (3110-3910)	3530 (3155-3890)		3520 (3130-3890)	3510 (3155-3910)	
1000 to <2500	55 (10.1)	50 (9.4)		57 (10.5)	48 (9.0)	
2500 to 3500	217 (40.0)	207 (38.8)		207 (38.0)	217 (40.9)	
>3500	271 (49.9)	276 (51.8)		281 (51.6)	266 (50.1)	
Infant sex:						
Male	325 (59.9)	336 (63.0)		336 (61.7)	325 (61.2)	
Female	218 (40.1)	197 (37.0)		209 (38.3)	206 (38.8)	
Any previous lumbar punctures	2 (0.4)	3 (0.6)		4 (0.7)	1 (0.2)	

Data are number (%) of participants, unless stated otherwise.

IQR=interquartile range.

### Explanation

Presenting baseline characteristics by treatment group is an essential part of reporting randomised trials. In factorial trials, this information might differ across main comparison, particularly for designs such as a partial factorial trial where eligibility criteria differ across factors. Presenting this information for each main comparison mitigates these issues.

## Participants numbers in analysis

- CONSORT 2010 item 16: For each group, number of participants (denominator) included in each analysis and whether the analysis was by original assigned groups
- Extension for factorial trials: For each main comparison, the number of participants (denominator) included in each analysis

CONSORT example: See figure 1.^53^

### Explanation

The denominator included in each treatment group is an essential part of the analysis. In factorial trials, the denominator might differ by the main comparison, and so presenting this information for each main comparison can increase clarity. This information is often included in a flow diagram but could be presented in other ways as well.

## Results

- CONSORT 2010 item 17a: For each primary and secondary outcome, results for each group, and the estimated effect size and its precision (such as 95% confidence interval)
- Extension for factorial trials: For each primary and secondary outcome, results for each main comparison, the estimated effect size and its precision (such as 95% confidence interval), for each primary outcome, the estimated interaction effect and its precision, and if done, estimated interaction effects and precision for secondary outcomes

CONSORT example: “At 3 years there was no evidence of a difference in bleeding on probing between randomised groups, for example, 0 versus 6-monthly: mean difference 0.87%, 95% CI [confidence interval]: −1.6 to 3.3, P=0.48 or between patients randomised to receive usual or personalised OHA [oral hygiene advice] (mean difference −2.5%, 95%CI: −8.3 to 3.3, P=0.39). … The interaction between personalised OHA and 6-monthly S&P [scale and polish] for bleeding was 1.7 (95% CI −3.8 to 7.3) (that is, neither statistically nor clinically significant).”^81^

### Explanation

Evaluation of statistical interactions is essential to the interpretation of most factorial trials, either because the validity of the design rests on the assumption of no interaction (2-in-1 trials) or the interaction itself is of main interest.^30^ ^33^ ^34^ ^39^ ^40^ ^41^ ^42^ The size of the estimated interaction effect should be presented for each primary outcome, and for secondary outcomes if done, along with a measure of precision such as 95% confidence interval.^34^ ^41^ ^42^ 2-in-1 trials are typically underpowered to identify all but very large interactions, and thus a large P value should not be taken as evidence that no interaction exists.^32^ ^42^

## Additional data summaries

- CONSORT 2010 item 18b: Additional data summaries (new item)
- New item for factorial trials: Participant flow, losses and exclusions, and outcome data (including primary and secondary outcomes, harms, and adherence) presented by treatment groups

**Fig 2:**
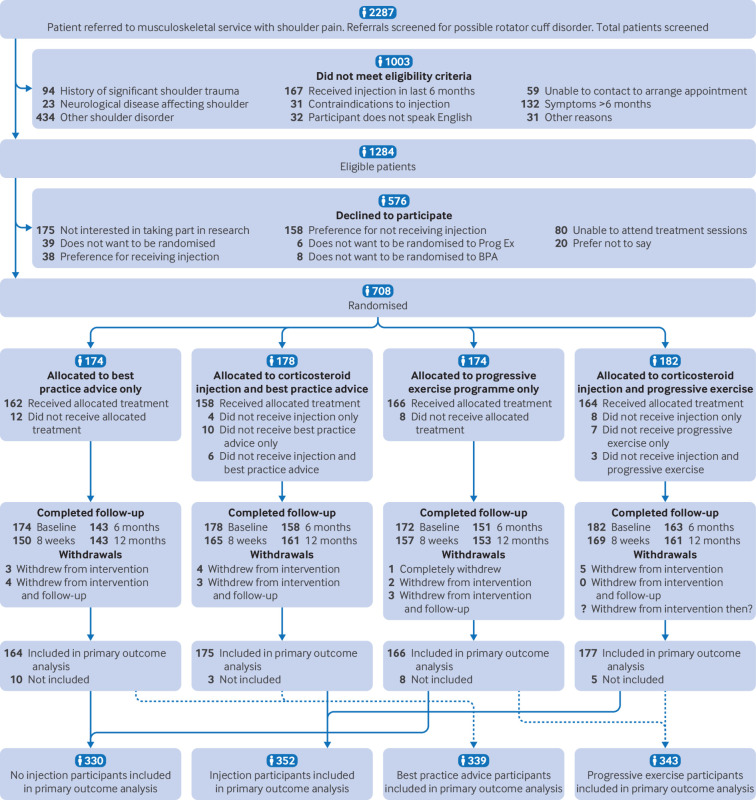
Example flow diagram of a factorial trial showing additional data summaries (GRASP trial). The GRASP trial compared progressive exercise (Prog Ex) with best practice advice (BPA) for patients with rotator cuff disorders. Figure adapted from Hopewell et al^51^

CONSORT example: See figure 2 and table 4.^51^

**Table 4:** Primary outcome data presented by treatment group in a factorial trial (GRASP). The GRASP trial compared progressive exercise with best practice advice for patients with rotator cuff disorders. Table adapted from Hopewell et al^51^

SPADI at each time point	Progressive exercise *v* best practice advice				Injection *v* best practice advice		
	Adjusted mean (SE), No of patients	Adjusted mean (SE), No of patients	Adjusted difference (99% CI)		Adjusted mean (SE), No of patients	Adjusted mean (SE), No of patients	Adjusted difference (99% CI)
8 weeks	41.22 (1.78), 156	41.09 (1.72), 149	−0.13 (−6.52 to 6.27)		32.89 (1.75), 163	37.97 (1.68), 149	−8.33 (−14.46 to −2.19)
6 months	26.99 (1.81), 151	25.71 (1.74), 143	−1.28 (−7.76 to 5.20)		27.75 (1.76), 158	26.02 (1.70), 143	0.76 (−5.45 to 6.97)
12 months	23.12 (1.81), 153	19.19 (1.74), 143	−3.93 (−10.40 to 2.55)		24.17 (1.76), 160	21.90 (1.71), 143	1.05 (−5.15 to 7.26)

CI=confidence interval; SE=standard error; SPADI=shoulder pain and disability index score.

### Explanation

Allocation to additional factors in factorial trials could affect outcomes and other post-randomisation data such as adherence, harms, and participant flow. For instance, receipt of factor B might reduce adherence to factor A compared with participants not allocated to factor B.^24^ Therefore, presentation of such data by treatment group (ie, groups A alone, B alone, A+B, and double control), in addition to presentation by main comparisons, allows readers to assess how outcomes and process variables might be influenced by the factorial design.^21^ ^30^ ^38^ ^39^ ^41^ ^42^ Furthermore, the presentation of such data could be essential to inform future meta-analyses of specific treatment comparisons (eg, A+B *v* B alone), which would not be available from standard factorial analysis comparisons.

Most trials report participant flow using a CONSORT flow diagram.^3^ Sometimes participant flow across each factor and across the treatment groups can be combined into a single diagram.^82^ However, this approach can be challenging with more complex factorial designs, or when the flow diagram requires large amounts of information for each group. An alternative way to report this information is to present separate diagrams using the existing CONSORT template, one describing participant flow for each factor, and one describing participant flow across the treatment groups. In a standard 2×2 factorial design, this would encompass three diagrams (one for factor A, one for factor B, and one for the four treatment groups). If a journal has space constraints, presentation of the additional diagrams may be added to the supplementary appendices.

Similar considerations exist for reporting other outcome data. For a standard 2×2 factorial design, data could be reported using three separate tables (one for factor A, one for factor B, and one for the four treatment groups), with some tables being added to the supplementary appendices due to space constraints.

## Harms

- CONSORT 2010 item 19: All important harms or unintended effects in each group (for specific guidance, see CONSORT for harms)
- Extension for factorial trials: All important harms or unintended effects for each main comparison

CONSORT example: See table 5.^53^

**Table 5:** Safety outcomes in a factorial trial (NeoCLEAR). The NeoCLEAR trial assessed infant position and timing of stylet removal to improve lumbar puncture success in newborn babies. Table adapted from Marshall et al^53^

Outcome	Sitting (n=543)	Lying (n=533)	Adjusted effect estimate (95% CI)	Early removal (n=545)	Late removal (n=531)	Adjusted effect estimate (95% CI)
Procedure abandoned due to cardiorespiratory deterioration (first procedure) (No (%))	2 (0.4)	1 (0.2)	Risk ratio 1.96 (0.17 to 22.08)	1 (0.2)	2 (0.4)	Risk ratio 0.49 (0.04 to 5.53)
Procedure abandoned due to cardiorespiratory deterioration (second procedure) (No/total No (%))	0/76 (0.0)	1/90 (1.1)	—	0/81 (0.0)	1/85 (1.2)	—
Infant’s lowest oxygen saturation (first procedure) (%; median (IQR))	93 (89-96)	90 (85-94)	Median difference 3.0 (2.1 to 3.9)	92 (86-95)	92 (87-95)	Median difference 0.0 (−0.9 to 0.9)
Infant’s lowest heart rate (first procedure) (bpm; mean (SD))	129.5 (19.9)	127.0 (21.5)	Mean difference 2.5 (0.6 to 4.4)	128.1 (21.0)	128.4 (20.4)	Mean difference −0.3 (−2.3 to 1.7)
Infant’s highest heart rate (first procedure) (bpm; mean (SD))	163.7 (21.7)	163.6 (21.9)	Mean difference 0.1 (−2.1 to 2.4)	163.9 (21.6)	163.4 (22.0)	Mean difference 0.5 (−1.9 to 2.9)
Respiratory deterioration post-LP (requirement for escalating respiratory support within 1 h of LP) (first procedure) (No (%))	1 (0.2)	2 (0.4)	Risk ratio 0.49 (0.04 to 5.71)	1 (0.2)	2 (0.4)	Risk ratio 0.49 (0.04 to 5.63)
Respiratory deterioration post-LP (requirement for escalating respiratory support within 1 h of LP) (second procedure) (No/total No (%))	0/76 (0.0)	0/90 (0.0)	—	0/81 (0.0)	0/85 (0.0)	—

Bpm=beats per minute; IQR=interquartile range; LP=lumbar puncture; SD=standard deviation.

### Explanation

A summary of harms or unintended effects is essential to help readers understand both the benefits and drawbacks of an intervention. In factorial trials, this approach might differ by the main comparison, and so presenting this information for each main comparison can increase clarity.

## Conclusions

Factorial trials offer the opportunity to efficiently answer multiple research questions in a single study, or alternatively to answer questions that cannot be investigated in standard parallel group designs such as whether treatments interact. However, factorial trials involve additional complexity in their design, conduct, analysis, and interpretation, which needs to be appropriately handled and reported. Reviews of factorial trials have identified major deficiencies in both reporting and methodology. The CONSORT 2010 and SPIRIT 2013 extensions discussed here^48^ ^49^ provide guidance for investigators to improve the reporting of both protocols and trial reports. This explanation and elaboration article provides details and examples to help authors implement these extensions.

## Notes

Contributors: BCK and EJ contributed equally; DE and AM contributed equally. BCK, SH, EB, A-WC, DE, EJ, and AM participated in the concept and design of the work. All the authors participated in the acquisition, analysis, and interpretation of data, and in the critical revision of the manuscript for important intellectual content. BCK, SH, and EB gave administrative, technical, and material support. BCK is guarantor. The corresponding author attests that all listed authors meet authorship criteria and that no others meeting the criteria have been omitted.

Provenance and peer review: Not commissioned; externally peer reviewed.
